# Nucleoside-Diphosphate-Kinase of *P. gingivalis* is Secreted from Epithelial Cells In the Absence of a Leader Sequence Through a Pannexin-1 Interactome

**DOI:** 10.1038/srep37643

**Published:** 2016-11-24

**Authors:** Kalina Atanasova, Jungnam Lee, JoAnn Roberts, Kyulim Lee, David M Ojcius, Özlem Yilmaz

**Affiliations:** 1 Department of Periodontology, University of Florida, Gainesville, Florida, USA; 2 Department of Oral Health Sciences, College of Dental Medicine, Medical University of South Carolina, Charleston, South Carolina, USA; 3 Department of Biomedical Sciences, University of the Pacific, Arthur Dugoni School of Dentistry, San Francisco, USA; 4 Department of Microbiology and Immunology, College of Medicine, Medical University of South Carolina, USA

## Abstract

Nucleoside-diphosphate-kinases (NDKs) are leaderless, multifunctional enzymes. The mode(s) of NDK secretion is currently undefined, while extracellular translocation of bacterial NDKs is critical for avoidance of host pathogen clearance by opportunistic pathogens such as *Porphyromonas gingivalis. P. gingivalis*-NDK during infection inhibits extracellular-ATP (eATP)/P2X_7_-receptor mediated cell death in gingival epithelial cells (GECs) via eATP hydrolysis. Furthermore, depletion of pannexin-1-hemichannel (PNX1) coupled with P2X_7_-receptor blocks the infection-induced eATP release in GECs, and *P. gingivalis*-NDK impacts this pathway. Ultrastructural and confocal microscopy of *P. gingivalis*-co-cultured GECs or green-fluorescent-protein (GFP)-*P. gingivalis*-NDK transfected GECs revealed a perinuclear/cytoplasmic localization of NDK. eATP stimulation induced NDK recruitment to the cell periphery. Depletion of PNX1 by siRNA or inhibition by probenecid resulted in significant blocking of extracellular NDK activity and secretion using ATPase and ELISA assays. Co-immunoprecipitation-coupled Mass-spectrometry method revealed association of *P. gingivalis*-NDK to the myosin-9 motor molecule. Interestingly, inhibition of myosin-9, actin, and lipid-rafts, shown to be involved in PNX1-hemichannel function, resulted in marked intracellular accumulation of NDK and decreased NDK secretion from infected GECs. These results elucidate for the first time PNX1-hemichannels as potentially main extracellular translocation pathway for NDKs from an intracellular pathogen, suggesting that PNX1-hemichannels may represent a therapeutic target for chronic opportunistic infections.

Nucleoside-diphosphate-kinases (NDKs) are a family of multifunctional enzymes that are evolutionarily highly conserved among numerous species from bacteria to humans^1^,^2^. One of their main functions is to hydrolyze nucleotide triphosphates (NTP), such as ATP, by catalyzing the transfer of orthophosphate residues, and are essential for proper DNA synthesis and housekeeping of cellular functions^3^. NDKs are small (~11–18 kDa), mainly cytoplasmic proteins, usually forming polymers (tetra- or hexamers) and having no known signal leader sequence, thus currently are unassigned to a specific secretion pathway^2^,^3^,^4^. The significance of NDK, including the human NDK species, encoded by the *nm23* gene, has been suggested for the pathogenesis of several different forms of severe chronic diseases, including cancers^5^,^6^,^7^. In breast and other cancer types, the secretion of NDK from cancer cells has been shown to be critical for increased metastatic activity^8^,^9^. Additionally, the importance of NDK secretion for the establishment of persistent chronic infections by opportunistic pathogens has been well documented^1^,^5^,^10^,^11^,^12^,^13^,^14^,^15^,^16^,^17^. Although NDKs have been shown to be secreted by the bacterial cells of a number of microbial species, the specific pathways of bacterial secretion, as well as their secretion from infected host cells, remain to be determined^1^. In the context of host infection, a number of microbial homologues of NDK have been identified, with either similar or diverse functions^11^,^12^,^13^,^14^,^15^,^16^,^17^. For example, during *Mycobacterium tuberculosis* infection, its NDK homolog has been found to help immune evasion by modulating apoptosis in macrophages, and by inactivating host small GTPases involved in free-radicals production and thus rescuing the bacteria from intracellular killing^11^,^12^,^13^. Another function of NDK has been linked to inhibition of NADPH-oxidase complex mediated bacterial killing during infections by *Burkholderia species* and *Porphyromonas gingivalis*^1^,^14^,^15^. Further, other observed multifactorial properties of bacterial NDKs include inhibition of danger-signal-eATP/P2X_7_-receptor mediated host cell and the bacterial killing as it has been also demonstrated in the opportunistic oral pathogen *Porphyromonas gingivalis* and lately in infections with *Chlamydia trachomatis,* or with the protozoan *Leishmania amazonensis,* thus contributing to the pathogens’ survival^1^,^10^,^16^,^18^.

It has been revealed that NDK is a critical virulence factor for *P. gingivalis*, to successfully survive in epithelial tissues by decreasing eATP concentration and thus hindering the downstream activation of eATP/P2X_7_-receptor mediated host-signaling pathways^5^,^18^,^19^,^20^. Our previous studies have shown that *P. gingivalis*-NDK is secreted outside of host cells during the infection of primary gingival epithelial cells (GECs) in a time-dependent manner^1^,^19^,^21^. It was also shown that *P. gingivalis*-NDK extracellular translocation from host cells did not result from damaged or compromised host cell membranes or host cell death^1^,^18^,^19^,^22^,^23^. The secreted enzyme was biologically active and functional, and its enzymatic activity was similar to the observed hydrolysis activity reported for MgsA (a homologue of the human AAA+ family proteins) in *Escherichia coli*^21^. The extracellular translocation of *P. gingivalis*-NDK from GECs was shown to modulate and inhibit key immune responses, such as eATP-induced NADPH-oxidase, as well as mitochondria-mediated reactive oxygen species (ROS) generation mediating oxidative stress and clearance of intracellular bacteria^1^,^18^,^21^,^24^. Secretion of *P. gingivalis*-NDK also interferes with eATP/P2X_7_-receptor mediated activation of the NLRP3 inflammasome in GECs, and suppresses the secretion of a pro-inflammatory cytokine, interleukin-1β (IL-1β), via ATP scavenging^18^,^21^,^25^,^26^,^27^. Despite the emerging importance of NDK secretion in a number of severe human chronic conditions, currently the extracellular translocation pathways of NDKs from host cells have not been identified^1^,^5^,^8^,^9^. Our recent studies have indicated that eATP release from *P. gingivalis*-infected GECs is largely mediated by the Pannexin-1-hemichannel (PNX1). The current literature has suggested that PNX1-hemichannel associates with the P2X_7_-receptor to form a functional complex, mediating the eATP-induced inflammasome activation and IL-1β secretion from danger-stimulated and infected cells^25^,^28^. Similarly to NDKs, IL-1β does not contain any known signal leader sequence for classical membrane trafficking and secretion pathway(s), but can be translocated extracellularly from host cells either upon infection or inflammatory stimuli^29^. PNX1-hemichannel activation was shown to play a major role for the extracellular release of ATP from GECs^21^. We have earlier also shown that depletion of PNX1 via siRNA can inhibit the eATP release from *P. gingivalis*-infected primary GECs, as well as impact the eATP-mediated ROS production and subsequent intracellular bacterial killing of *ndk*-deficient mutant strain of *P. gingivalis*^1^,^18^. Taken together, accumulated evidence suggests that PNX1-hemichannel may be utilized specially by *P. gingivalis* for the release of NDK from inside to outside of the infected cells.

Our results from this study demonstrate that during infection, *P. gingivalis*-NDK can be detected in the cytoplasm of infected cells, both ultrastructurally by transmission electron microscopy (TEM) and by immunofluorescence microscopy, predominantly in the perinuclear area. Interestingly, the mobilization of NDK to the cell periphery appears to be activated upon eATP-stimulation of GECs. Inhibition of PNX1-hemichannel either by siRNA or via the pharmacological inhibitor, probenecid, substantially reduced the secretion of NDK outside of the host cells. The lipid-rafts inhibitor methyl-β-cyclodextrin (MβCD), in conjunction with PNX1-hemichannel blocking, also inhibited the extracellular translocation of *P. gingivalis*-NDK. Mass spectrometry of proteins co-immunoprecipitated with the NDK from *P. gingivalis-*infected GECs revealed a specific association of the NDK with the non-muscle Myosin-9 host cell motor molecule, which suggested myosin-dependent cellular transport to the cell membrane. Myosin-9 is known to bind to actin during the formation of the intracellular trafficking machinery, suggesting the potential coupling of the actin cytoskeleton to the extracellular translocation of *P. gingivalis*-NDK^30^. The specific pharmacological inhibition of Myosin-9 through the ML9 inhibitor and of actin polymerization using cytochalasin D, also reduced significantly the secretion of NDK from *P. gingivalis*-infected GECs, thus displaying the joint involvement of Myosin-9 and actin in this specific process. Hence, this study suggests for the first time the utilization of PNX1-hemichannel, Myosin-9 and the actin interactome, as a transcellular transport mechanism by a microbial NDK. Accordingly, the PNX1-hemichannel secretion pathway may represent a potential target for the control of *P. gingivalis* persistence in the epithelial tissues of the oral cavity.

## Results

### Transmission electron microscopy (TEM) analysis of NDK localization in primary GECs

**Figure 1:**
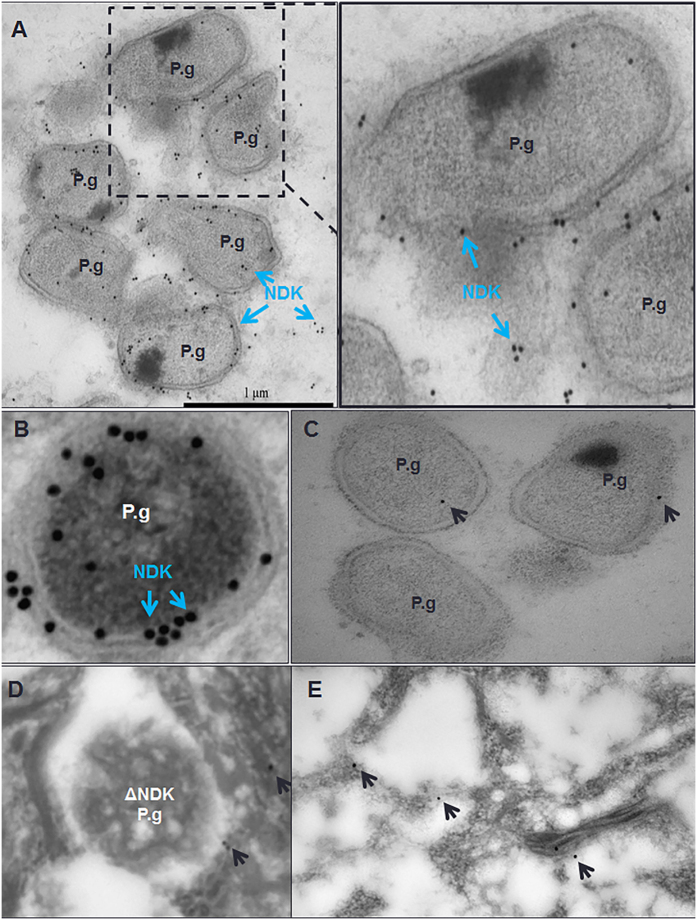
Nucleoside diphosphate kinase (NDK) secretion from *P. gingivalis* (P.g.) is shown both in the cytoplasm of the infected host cells and on the surface of *P. gingivalis* bacteria at 12 hours after infection in gingival epithelial cells (GECs). NDK was visualized by transmission electron microscopy using immunogold labelling and rabbit anti-*P. gingivalis* NDK antibody. NDK (blue arrows) is seen on the *P. gingivalis* bacterial surface and in the host cytoplasm independently of the bacteria (**A,B**). An enlarged image of the boxed area is shown to the right (**A**). *P. gingivalis* with no primary antibody incubation (**C**), *ndk-*deficient mutant strain, ΔNDK (**D**) and GECs without infection (**E**) with both primary and secondary antibody incubations were used as controls. The black arrows point to non-specific background level of gold labelling staining in the control samples. Bar represents 1 μm.

Our previous studies revealed that NDK exhibits a time dependent increase with steady enzyme kinetics in secretion from infected GECs, and was also detected in the soluble and insoluble fractions of those cells using Western blot analysis^21^. We wanted to utilize TEM analysis, by using *P. gingivalis*-NDK specific polyclonal antibody recognized by immuno-gold labelled detection system, to visually identify and locate *P. gingivalis*-NDK within infected GECs. *P. gingivalis* NDK was observed both on the surface of the intracellularly situated bacteria and freely in the host cytoplasmic space independent of bacterial location (Fig. 1A and B). *P. gingivalis* infected GECs lacking primary antibody and probed with gold-labelled secondary antibody (Fig. 1C) or GECs infected with the *ndk*-deficient mutant strain (Fig. 1D) or GECs with no infection (Fig. 1E) conditions were used as controls which revealed only very little non-specific background staining further verifying the positive results shown in Fig. 1A and B.

### Localization of P. gingivalis-NDK in infected or transfected primary GECs upon eATP-stimulation

**Figure 2:**
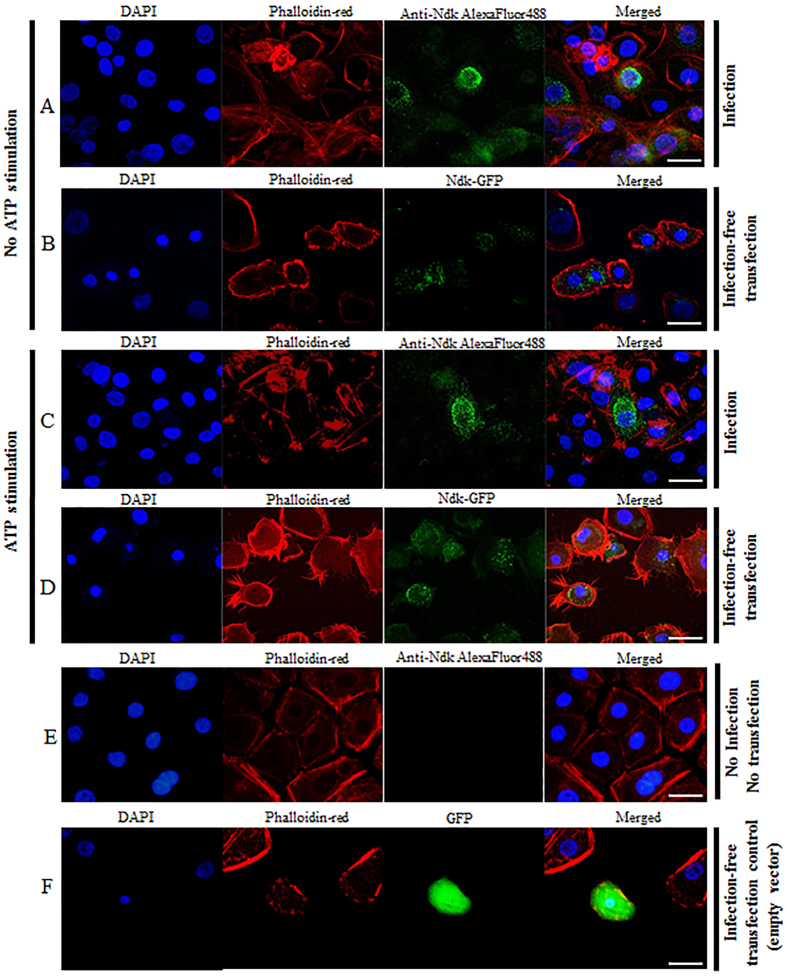
Immunofluorescence analysis of *P. gingivalis*-NDK cellular localization in GECs. (**A**) 12 h of *P. gingivalis* infection; (**B**) transfection with GFP-linked *P. gingivalis*-NDK. (**C**) 3 mM ATP stimulation of 12 h *P. gingivalis*-infected GECs; *P. gingivalis*-NDK in infected cells (**A,C**) was detected using rabbit anti-*P. gingivalis* NDK antibody and visualized with anti-rabbit AlexaFluor488 secondary antibody; (**D**) 3 mM ATP of GECs transfected with GFP-linked *P. gingivalis-*NDK (**E**) No-infection, no-transfection control cells showed no unspecific staining of NDK. (**F**) Transfection with GFP-containing plasmid, without *P. gingivalis*-NDK insertion (empty vector), showed an unspecific uniform GFP expression throughout the transfected cells as expected. These are representative images of at least three separate experiments performed in duplicates. All images represent maximum image projections of z-sections performed on laser confocal microscope. Bars represent 15 μm.

The NDK species have been suggested to be cytoplasmic proteins^1^,^3^. Accordingly, we aimed to further examine the subcellular localization of *P. gingivalis*-NDK within host cells using confocal fluorescence microscopy, which revealed that *P. gingivalis*-NDK was primarily found in the perinuclear area of the infected GECs, and some in the cytoplasm (Fig. 2A). We also utilized a green-fluorescent-protein (GFP) construct of *P. gingivalis*-NDK, and introduced it into primary GECs by transfection (Fig. 2B and D). This approach was taken to verify whether the cytoplasmic localization is dependent on the presence of whole bacteria^3^. Similarly, the GFP-NDK transfected GECs also displayed localization of NDK largely in the perinuclear and some in the cytoplasmic area (Fig. 2B). Since we previously showed that *P. gingivalis* infection induces the release of ATP from infected GECs, and that *P. gingivalis* secretes NDK to modulate host cell death mediated by eATP during the infection, we investigated the potential effect of eATP treatment on NDK trafficking in GECs. Interestingly, eATP treatment of GECs induced NDK translocation towards the cell periphery in both the infected and the GFP-NDK-transfected (infection-free) cells (Fig. 2C and D), suggesting that NDK enzyme translocation is likely activated by eATP-stimulation. A quantitative analysis of NDK’s subcellular localizations through fluorescence intensity measurements using NIH image analysis further supported the mobilizing effect of eATP on the cytoplasmic NDK (Supplementary Fig. 1).

### Secreted NDK from infected GECs is biologically active and can be inhibited via PNX1 inhibition

**Figure 3:**
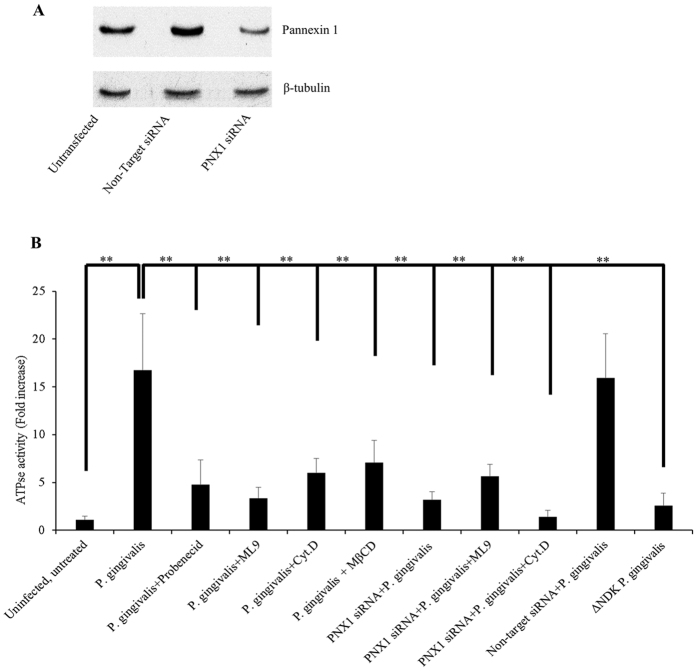
Detected NDK enzyme ATPase activity in cell culture media of *P. gingivalis*-infected GECs in the presence or absence of PNX1 or lipid raft inhibition. (**A**) A representative image of the expression of the 45 kDa PNX1 protein in PNX1 knock-down GECs at 48 h post transfection, as detected by Western blot assay; β-tubulin (51 kDa) was used as loading control; Full-length blots are presented in Supplementary Figure 2; (**B**) GECs were infected with *P. gingivalis* and treated with inhibitors of PNX1 (probenecid), Myosin-9 (ML9), an actin cytoskeleton inhibitor (cytochalasin D), or a lipid raft inhibitor (MβCD), or were transfected with PNX1 siRNA and infected with *P. gingivalis* in combination with treatment with ML9 or cytochalasin D. *ndk*-deficient mutant strain of *P. gingivalis* was used as control. All data represent an average of at least three separate experiments. P-values were calculated using a two-tail student *t*-test. ** Represent P-values < 0.001.

Because ATP appears to be an important stimulus for NDK translocation, and our previous studies demonstrated that ATP release is mainly mediated through the PNX1-hemichannel in GECs and *P. gingivalis*-NDK specifically impacts the PNX1- pathway^1^,^21^, we examined the potential significance of PNX1-hemichannel for the secretion of the small effector NDK from infected GECs. Simultaneously, we studied whether the secreted NDK is active and functional. Therefore, we first performed a series of ATPase activity assays on *P. gingivalis*-infected GECs treated with either a PNX1 inhibitor (probenecid), or an inhibitor of lipid-rafts (MβCD), which are shown to play a role in the regulation of PNX1-hemichannel activity^31^,^32^,^33^. We also utilized siRNA technology to knock down the PNX1-hemichannel to further examine the functional role of PNX1. PNX1-hemichannel depletion was verified by both quantitative real-time PCR (data not shown) and Western blot analyses and an average reduction of ≥70% in PNX1 expression was confirmed by both techniques (Fig. 3A). The extracellular media of *P. gingivalis*-infected GECs showed significantly high ATPase activity, compared to uninfected GECs and the *ndk*-deficient mutant strain infected GECs, altogether confirming that the secreted NDK is an active enzyme (Fig. 3B). Both probenecid and MβCD treatments significantly reduced the ATPase activity in the infected cells’ culture media (P values for both inhibitors were <0.00005), when compared to infected cells without inhibitors (Fig. 3B). PNX1 knock down of infected cells via siRNA showed more than 50% reduction of ATPase activity (P value < 0.00005) in the collected cell media, when compared to the infected cell media without PNX1 depletion (Fig. 3B). Overall these results point out possibly an important role of PNX1-hemichannels for the secretion of *P. gingivalis*-NDK outside of infected GECs.

### Mass spectrometry analysis of NDK molecule interactors for intracellular trafficking

NDK species in general have been suggested to have multiple functions intracellularly and can work directly or indirectly with a number of components of the host cell including membrane trafficking, and energetics system^1^,^3^,^5^,^7^,^34^. Therefore we examined whether we can identify a cellular cytoplasmic molecule that can interact with *P. gingivalis*-NDK and facilitate the intracellular trafficking of the enzyme to the cell membrane and perhaps outside the host cells. Accordingly we performed a liquid-chromatography-tandem-mass-spectrometry analysis of the proteins co-immunoprecipitated with *P. gingivalis*-NDK within infected GECs, which revealed a strongest binding of Myosin-9 (Cluster of Isoform-1, IPI00019502) to NDK, with over 95% specificity and over 99.9% threshold in the detection, at a setting of 5 or more matching peptides^35^,^36^. This result suggested a putative role of Myosin-9 in this specific interaction. Pairing of Myosin-9 motor molecule with NDK could thus be critical for the NDK transport, since previous studies demonstrated that Myosin-9 associates with actin as participants in the host cell trafficking systems, and both Myosin-9 and actin interact with PNX1-hemichannels^37^,^38^,^39^.

### Fluorescence microscopy analysis of NDK co-localization with Myosin-9

**Figure 4:**
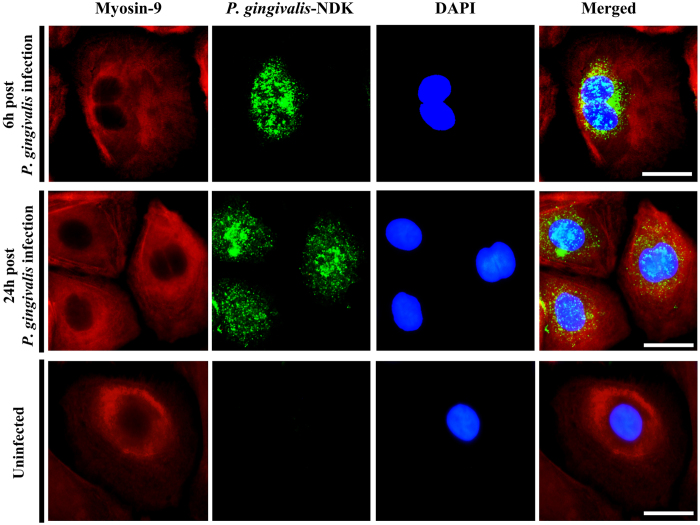
Confocal Fluorescence analysis of NDK and Myosin-9 co-localization in *P. gingivalis*-infected primary GECs **(Top)** at 6 h post infection, or (Middle) at 24 h post infection with *P. gingivalis*. Uninfected GECs were used as staining controls (Bottom). Myosin-9 was labelled in red by rabbit anti-human Myosin-9 monoclonal antibody, visualized with anti-rabbit AlexaFluor594 secondary antibody. *P. gingivalis*-NDK is labelled in green, detected by monoclonal *P. gingivalis*-NDK specific antibody, visualized with anti-mouse AlexaFluor488 secondary antibody. Cell nuclei were visualized with DAPI. Mander’s co-localization (yellow) coefficients for the two molecules ranged from 0.465 at 6 h to 0.791 at 24 h. Bars represent 5 μm.

To obtain further insight about the interaction between NDK and Myosin-9, we performed confocal fluorescence microscopy to analyze co-localization of NDK and Myosin-9 within infected cells at early stages of infection (6 h after infection), as well as at 24 h after infection, when NDK secretion has been observed to peak in primary GECs^1^,^19^,^21^. An increasing level of NDK/Myosin-9 co-localization was detected from 6 h to 24 h, with Mander’s coefficient of co-localization ranging from 0.465 at 6 h to 0.791 at 24 h (Fig. 4), further suggesting a time dependent significant increase in the interaction of NDK with Myosin-9.

### NDK secretion from infected GECs is mediated through PNX1, Myosin-9 and actin

**Figure 5:**
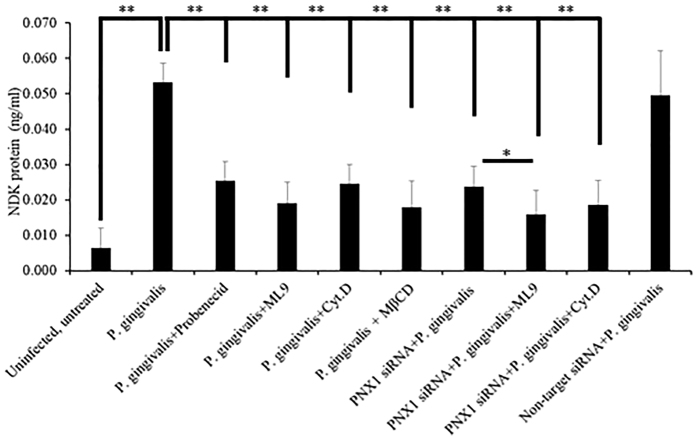
NDK secretion detected by ELISA in cell culture media of GECs infected with *P. gingivalis.* GECs were infected with *P. gingivalis* and treated with inhibitors of PNX1 (probenecid), Myosin-9 (ML9), an actin cytoskeleton inhibitor (cytochalasin D), or a lipid raft inhibitor (MβCD), or were transfected with PNX1 siRNA and infected with *P. gingivalis* in combination with treatment with ML9 or cytochalasin D. All data represent an average of at least three separate experiments. P-values were calculated using a two-tail Student *t*-test. ** and * represent P-values < 0.001 and P < 0.05 respectively.

Because Myosin-9 was previously shown to interact with the PNX1 membrane complex in the activation of ATP release and pore formation^38^,^40^, and actin has been shown to also associate with Myosin-9 and PNX1^38^,^41^,^42^,^43^,^44^, we examined whether Myosin-9 and actin, together with PNX1-hemichannels, can facilitate the extracellular secretion of *P. gingivalis*-NDK outside of the host cells. To examine the potential significance of Myosin-9 and actin, we measured the NDK secretion in the cell culture media of *P. gingivalis*-infected GECs, in the presence or absence of PNX1 depletion by siRNA or in the presence of probenecid (PNX1 inhibitor), ML9 (Myosin-9 inhibitor) or cytochalasin D (actin inhibitor). All three inhibitors, separately or jointly, significantly reduced the presence of *P. gingivalis*-NDK in the extracellular media (Fig. 5), compared to the untreated, *P. gingivalis*-infected GECs’ extracellular media (P values < 0.00005). PNX1 knock-down alone, or in combination with ML9 or cytochalasin D, also significantly reduced the presence of NDK protein in the extracellular media (Fig. 5), compared to the untreated, *P. gingivalis*-infected GECs (P-values < 0.00005), but the strongest reduction was observed in the PNX1 depleted cells treated with ML9 (P value = 1.8E^−27^) (Fig. 5). There was also a statistically significant difference for the extracellular NDK between PNX1 depleted infected cells and PNX1 depleted and ML9 treated infected cells (P value < 0.05). These results suggests that Myosin-9 association with PNX1 is likely critical for the NDK secretion from *P. gingivalis*-infected GECs. Furthermore, Myosin-9 and actin cytoskeleton, by associating with PNX1-hemichannels, could facilitate the *P. gingivalis*-NDK recruitment to the cell membrane and then secretion outside.

### Intracellular accumulation of NDK in infected GECs upon inhibition of PNX1, lipid-rafts, Myosin-9 or actin

**Figure 6:**
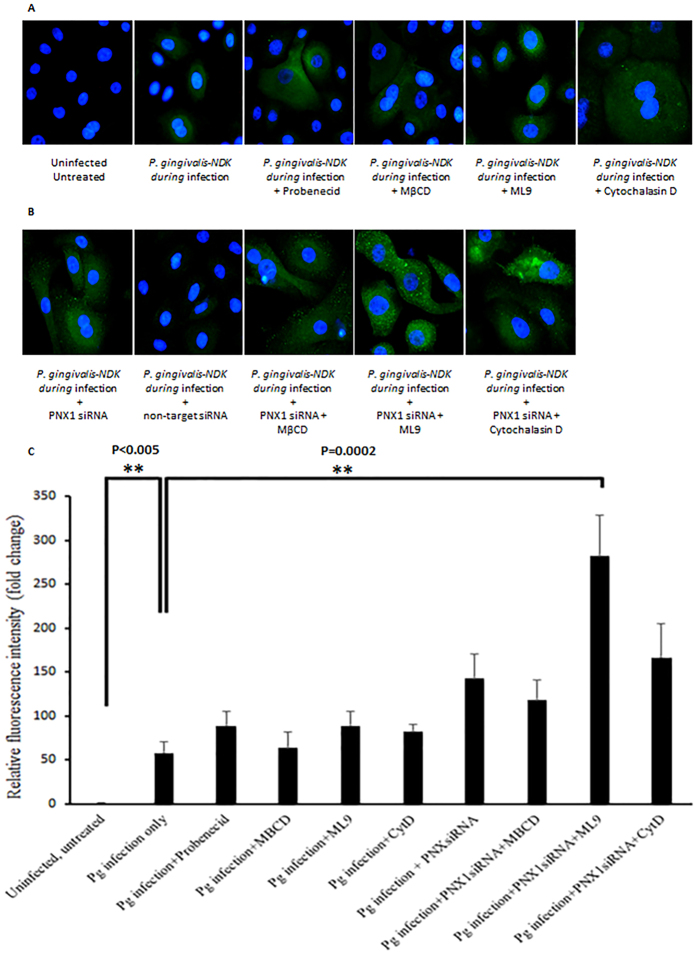
Immunofluorescence analysis of NDK intracellular accumulation. (**A**) *P. gingivalis*-infected primary GECs after treatment with probenecid, MβCD, ML9 or cytochalasin D. Uninfected, untreated cells were used as a control; **(B)** PNX1 siRNA-transfected *P. gingivalis*-infected primary GECs with or without MβCD, ML9 or cytochalasin D treatment. Non-target siRNA-transfected *P. gingivalis*-infected GECs were used as a control. *P. gingivalis*-NDK is labelled in green, detected by monoclonal *P. gingivalis*-NDK specific antibody, visualized with anti-rabbit AlexaFluor488 secondary antibody. Bar represents 1 μm. (**C**) NIH ImageJ analysis was performed for measuring cell fluorescent intensity. Cell boundaries were determined by actin labelling with phalloidin-TRITC. Corrected total cell fluorescence was calculated and measurements were normalized to the mean intensity of the uninfected, untreated cells. All data represent an average of at least three separate experiments. ** Denote P-values < 0.001. Select exact P-values are also shown.

Since our results revealed that inhibition of PNX1, along with Myosin-9 and actin cytoskeleton could prevent the extracellular secretion of *P. gingivalis*-NDK, we next examined whether the inhibition of the PNX1-hemichannel, as well as the blocking of putative coupling molecules that we identified in this study, Myosin-9, actin and lipid-rafts, can induce accumulation of *P. gingivalis*-NDK in the cells and subsequently inhibit the extracellular secretion. We employed fluorescence microscopy using *P. gingivalis*-NDK specific monoclonal antibody, to qualitatively observe increased intracellular fluorescence as a method to detect intracellular accumulation of *P. gingivalis*-NDK in infected primary GECs upon inhibition of PNX1, either by RNA silencing or by probenecid treatment, and treatment with MβCD, ML9 or cytochalasin D (Fig. 6A and B). All inhibitions, either alone or in combination, displayed increased *P. gingivalis*-NDK fluorescent staining in the PNX1 knock-down GECs. Most significant accumulation was present when we depleted PNX1-hemichannels via siRNA and treated with ML9 or cytochalasin D (Fig. 6B). A quantitative fluorescence intensity analysis shown in Fig. 6C substantiated the observed phenotypes in Fig. 6A and B and further demonstrated the conjoint significant involvement of ML9 and cytochalasin D in PNX1 knock-down GECs for extracellular mobilization of *P. gingivalis*-NDK. These findings collectively indicate that PNX1 hemichannels, along with the downstream partner molecules, such as Myosin-9 and the actin cytoskeleton, can mediate *P. gingivalis*-NDK transport to the cell membrane and later secretion outside of the host cells.

## Discussion

NDK enzymes were first discovered in the early 1950 s in yeast extract and pigeon muscle tissue^45^,^46^. Currently it is recognized that NDKs are present in a vast number of species from bacteria to human and they can perform multifunctional duties within the organism^1^,^2^,^34^. All presently sequenced and characterized NDKs represent tetra- or hexamers in their active enzyme form, with subunit size of 11–18 kDa^2^,^3^,^4^. Similar to other leaderless proteins that are shown to be secreted, there is still not much known on the specific pathways of NDK secretion, both on the microbial species level, as well as the higher organisms’ levels such as human^1^.

Our novel findings suggest that *P. gingivalis-*NDK can employ the PNX1 membrane hemichannel to be translocated outside of the host cells, which may in turn allow the bacteria to establish successful persistent infection within mucosal epithelial tissues. Although the activated PNX1-hemichannel/P2X_7_-receptor large-pore has been suggested to allow molecules of 1–1.5 kDa size^47^,^48^, the current knowledge on the mechanisms and function of this assembly, as well as generally on unconventional mechanisms of protein secretion, is still very incomplete. For example alternative pathways of secretion have been suggested to be involved in the extracellular secretion of the 17 kDa active form of IL-1β, possibly also involving the PNX1/P2X_7_-receptor large pore formation^31^, whereas the human 80 kDa fibroblast growth factor-2 seems to utilize direct transmembrane translocation^49^.

The results from our ultrastructural and immunofluorescence microscopy analyses demonstrate that during infection *P. gingivalis*-NDK can be detected in the cytoplasm of infected cells, largely in the perinuclear area, and its translocation to the cell membrane is augmented upon eATP stimulation (Supplementary Fig. 1). Moreover, our findings from GFP-*P. gingivalis*-NDK transfected cells indicate that the observed intracellular trafficking and secretion of NDK from infected GECs is a direct interaction between NDK and the host-cell transport molecules and is likely not dependent on other *P. gingivalis* effectors. Inhibition of the PNX1-hemichannels either by siRNA or via the pharmacological inhibitor, probenecid, significantly reduced the secretion of NDK outside of the host, suggesting the involvement of PNX1-hemichannel in the extracellular NDK translocation during infection.

Additionally, as expected due to the lack of known leader secretion motif, inhibition of Endoplasmic Reticulum-Golgi transport pathway did not affect the secretion of NDK protein (data not shown), further demonstrating that this enzyme does not utilize the classical host secretion pathways for translocation outside of infected cells. The mass-spectrometry analysis revealed a specific binding of *P. gingivalis*-NDK with the host-cell non-muscle Myosin-9 motor molecule, which suggested myosin-associated transport through the cell membrane. Confocal immunofluorescence co-localization analysis of NDK and Myosin-9 in *P. gingivalis*-infected GECs at early (6 h post infection) and peak (24 h post infection) NDK secretion stages revealed an increasing high level of co-localization between NDK and Myosin-9, also supporting the involvement of Myosin-9 in NDK trafficking outside of the host cells. Consistent with those results, the employment of Myosin-9 specific inhibitor ML9 which had a large inhibitory effect on NDK secretion alone (Figs 3, 5 and 6), significantly enhanced the inhibition of NDK secretion from PNX1 depleted *P. gingivalis* infected cells (Figs 3, 5 and 6), further inferring the partnership of PNX1 with Myosin-9 in the NDK trafficking to extracellular space.

These findings were substantiated by previous studies proposing binding and complex association of non-muscle myosin with P2X_7_-receptor and PNX1-hemichannel in the membrane complex^38^,^41^. Furthermore, non-muscle myosins, and especially Myosin-9, have been shown to bind to actin filaments and to play an essential role during the formation and function of multiple intracellular trafficking, secretory and organellar positioning machineries^30^,^50^,^51^, proposing also the involvement of the actin cytoskeleton in the translocation of *P. gingivalis*-NDK outside of host cells. Actin polymerization indeed has been demonstrated to play a key role in the intra- and inter-cellular translocation of *P. gingivalis* in a time dependent manner^19^,^52^,^53^,^54^,^55^. Moreover, a human homologue of non-muscle myosin was found to form a complex with the non-ATP-stimulated P2X_7_-receptor protein complex in THP-1 and HEK-293 cell lines, and the NDK dissociated from the complex upon ATP stimulation which may facilitate its secretion outside of the stimulated cells^38^. This process was also associated with the regulation of the pore formation^38^.

PNX1 has been closely linked with several inflammatory conditions and chronic diseases including several types of cancers (keratinocyte-derived basal cell carcinoma, squamous cell carcinoma and glioma), gout, urinary bladder, skin and brain conditions^7^,^39^. Moreover, PNX1-hemichannels have been associated with increased metastasis in cancers, and human NDK species (NME/NM23 Nucleoside Diphosphate Kinase 1) have been also shown to play a crucial role in cancer metastasis^8^,^9^ Recently, the PNX1 inhibitor probenecid has been suggested for treatment of several inflammatory diseases including bladder inflammation-related increased blood levels of uric acid, and is undergoing multiple clinical trials for other conditions^56^.

**Figure 7:**
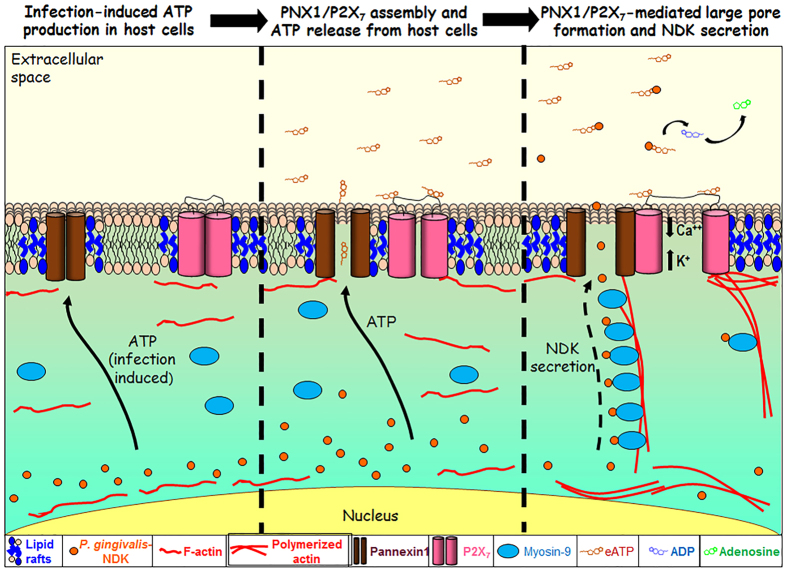
A proposed pathway of NDK secretion from infected host cells. Upon infection with *P. gingivalis,* an initial activation of ATP release from the cell arises, and the P2X_7_-receptor/Pannexin-1 (PNX1)-hemichannel is activated^1^,^21^,^25^. *P. gingivalis*-NDK is accumulated in the cytoplasm and mostly in the perinuclear area. The accumulated *P. gingivalis*-NDK is activated upon ATP release, which can act as an autocrine danger signal to the host by stimulating the P2X_7_-receptor/PNX1-hemichannel. Following activation, *P. gingivalis*-NDK interacts Myosin-9 motor molecule and is trafficked along the Myosin-9 and actin filaments to the cell periphery. NDK translocates to the extracellular space through the forming P2X_7_-receptor/PNX1 channel. Secreted NDK hydrolyzes the danger-signal eATP, thus attenuating the stimulation of the P2X_7_-receptor/PNX1-hemichannel complex, and the downregulating the associated downstream events, such as reactive oxygen species production and intracellular bacterial killing^1^,^18^,^19^,^21^,^25^.

In light of our results and the currently available literature, we propose a novel molecular pathway for *P. gingivalis*-NDK extracellular translocation (Fig. 7). Our study elucidates for the first time a potential shared mechanism of unconventional translocation of intracellular NDKs outside of host cells, involving PNX1/P2X_7_-complex-mediated large pore formation. We also realize that NDKs might have multiple redundant pathways for exhibiting their function and biology varying from one organism to another. Our findings may have implications in various fields of research, from basic bacterial-host interaction and possible control of persistent intracellular bacterial infections, to management of some NDK secretion and PNX1- related chronic conditions. The elucidated molecular mode of action can also be a valuable contribution to future studies designed to dissect the importance of PNX1-hemichannel and *pnx1-*allelic variations in other chronic diseases.

## Experimental procedures

### Bacterial strains, eukaryotic cells, media and reagents

*P. gingivalis* ATCC 33277 strain and its isogenic *ndk*-deficient mutant strain were cultured to mid-log phase in Tripticase soy broth (TSB) supplemented with yeast extract (1 ug/ml), menadione (1 ug/ml) and hemin (5 ug/ml), at 37 °C under anaerobic conditions and harvested as described previously^22^. Erythromycin (10 mg/ml) was added to the media as a selective agent for the growth of the mutant strain, which was previously described^18^. The number of bacteria for infection was determined using a Klett-Summerson photometer (Klett). Bacterial cells were used at a multiplicity of infection (MOI) of 100 in all infection experiments. Primary cultures of gingival epithelial cells (GECs) were generated and cultured as described previously^18^,^57^. No subject recruitment per se was done. Adult patients were selected at random and anonymously from those presenting at the University of Florida Dental Clinics for tooth crown lengthening or impacted third molar extraction. No patient information was collected. Gingival tissue that would otherwise be discarded was collected after informed consent was obtained by all patients under the approved guidance of the University of Florida Health Science Center Institutional Review Board (IRB). All experimental protocols and all methods were approved by University of Florida IRB committee and were carried out with all applicable federal regulations governing the protection of human subjects.

### Transmission electron microscopy with immunogold labeling

GECs were seeded onto coverslips and when at 70% confluence cells were co-cultured with *P. gingivalis* ATCC 33277 strain or the *ndk*-deficient mutant strain for 12 hours. Specimens were then harvested and placed in 1.5% paraformaldehyde/0.025% glutaraldehyde solution for 1 h, and then in phosphate buffer. The fixed specimens were dehydrated using a graded ethanol series; cells were incubated in different ratios of 85% ethanol: LR White embedding media as outlined elsewhere^58^. Samples were then allowed to incubate 1 h in 100% LR White followed by a fresh exchange and overnight incubation at 4 °C. The following day, specimens were incubated in fresh LR White for 1 h, placed in gelatin capsules, centrifuged (1500 × G, 5 min) and the blocks polymerized by incubation for 20–24 h at 58 °C. Ultrathin sections were mounted onto nickel grids and blocked with normal goat serum diluted 1:100 for 1 h. Each grid was incubated with a 1:500 dilution of anti-*P. gingivalis* 33277 NDK rabbit polyclonal antibody for 75 minutes. Gold-conjugated secondary antibodies were used at a 1:20 dilution with 1 h incubation. Samples were imaged using a Tecnai BioTwin (FEI Company, Hillsboro, OR, USA) electron microscope operating at 80 or 120 kV. Digital images were captured using a 2 K × 2 K camera (AMT, Danvers, MA, USA). For gold-label enumeration, control grids of uninfected cells with primary and secondary antibodies or with secondary antibody alone were incubated and background levels of labeling were quantified. Compartments with greater than background levels of antibody binding were scored as positive. Accordingly, each grid of infected cells was scored for positive labeling based on control experiments.

### Fluorescent microscopy

#### Confocal microscopy

GECs were seeded at a density of 8 × 10^4^ on glass coverslips (Warner Instruments) in four-well plates (Thermo Fisher Scientific) and cultured until ~70% confluence. Cells were infected with *P. gingivalis* ATCC 33277 and incubated for a 24 h time period. Some wells were first infected with *P. gingivalis* ATCC 33277 and then 1 h later were treated with 3 mM ATP until collection. Cells were fixed with 10% neutral buffered formalin (NBF), permeabilized by 0.1% Triton X-100, and stained for 1 h at room temperature with a rabbit polyclonal antibody raised against *P. gingivalis* ATCC 33277 nucleoside diphosphate kinase, NDK (GenScript). The stained cells were washed and incubated for 1 hour at room temperature with Alexa Fluor 488 conjugated secondary goat anti-rabbit polyclonal antibody (1:1000; Invitrogen). For labeling of actin-cytoskeleton phalloidin-tetramethylrhodamine β-isothiocyanate (TRITC) was used (Sigma-Aldrich). Coverslips with fixed cells were mounted onto Corning glass microscopy slides using VectaShield mounting medium containing DAPI (Vector Laboratories). Images were acquired using LSM710 confocal microscope (Zeiss) using the Zen 2011 software.

For the GFP-NDK experiments GECs were seeded and cultured until a confluence of ~85%, at which point cells were transfected with a pEGFP-C1 vector (gift from Dr. Fredrick Southwick) transformed with a *P. gingivalis* ATCC 33277 NDK insert (gene PGN-1337) using Lipofectamine 2000 reagent (Invitrogen) per manufacturer’s instructions. pEGFP-C1 vector, not containing an insert (empty vector) was used as control for the specificity of the localization and expression. Transfected GECs were cultured for additional 48 hours, and some wells were treated at 36 h after transfection with 3 mM ATP for 12 h before the collection. All cells were collected, stained and visualized as described in the first set of experiments above.

Measurement of relative fluorescence intensity was performed using NIH ImageJ software. Cell boundaries were determined from the actin cytoskeleton staining (phalloidin-TRITC, staining red). Mean fluorescence, cell area and the integrated density for each cell were measured by the software. The Corrected Total Cell Fluorescence (CTCF) was calculated as follows: CTCF = Integrated density − (Area of selected cell × Mean fluorescence of background readings). A minimum of 15 high magnification images of cells, originating from at least 3 separate experiments, were evaluated for each experimental condition. For some of the analyses the fluorescence intensity of the peripheral cytoplasmic area was calculated as the intensity of the whole selected cell minus the intensity of the perinuclear area.

#### Epifluorescence microscopy

GECs were seeded at a density of 8 × 10^4^ on glass coverslips (Warner Instruments) in four-well plates (Thermo Fisher Scientific) and cultured until ~70% confluence. Cells were infected with *P. gingivalis* ATCC 33277 and incubated for a 12 h time period. Some wells were first infected with *P. gingivalis* ATCC 33277 at an MOI of 100 and 1 h later were treated with probenecid (PNX1 inhibitor, Sigma-Aldrich) at final concentration of 1 mM; methyl-β-cyclodextrin (Lipid-rafts inhibitor, Sigma-Aldrich) at final concentration of 10.5 μM; ML9 (Myosin-9 inhibitor, Sigma-Aldrich) at final concentration of 30 μM; or cytochalasin D (inhibitor of actin polymerization, Sigma-Aldrich) at final concentration of 1 ug/ml.

For the knockdown experiments, GECs seeded on glass coverslips were first transfected with a mixture of 10 pmol of PNX1-targetting short interfering RNA (siRNA) (Life Technologies, Silencer Select) and 3 μl/ml Lipofectamine RNAiMax reagent (Life Technologies), according to the manufacturer’s instructions. A non-targeting control siRNA was used as reference for every condition (Life Technologies, Silencer Select). 48 hours after transfection cells were infected with *P. gingivalis* ATCC 33277 at an MOI of 100 and incubated for a 12 h time period. 1 hour after infection cells were treated with the same inhibitors at same concentrations as described in the above experiments. *P. gingivalis* was co-cultured with the host cells for 1 hour before the host cells were treated with cytochalasin D or other agents including the lipid rafts inhibitor. The intracellular entry of *P. gingivalis* ATCC 33277 into human primary GECs has been shown to be swift dynamic process where vast majority of the bacteria were found to be intracellular following 20 minutes of incubation^52^,^55^. Accordingly, actin cytoskeleton inhibitor (cytochalasin D) and all the other inhibitors were employed at 1 hour post infection when the internalization of *P. gingivalis* was complete. All the used inhibitor concentrations were selected on basis of previous literature and a series of experiments performed in this study which showed no effect on bacterial or cellular viability for the experimental duration^52^.

All cells from both sets of experiments were fixed with 4% paraformaldehyde, permeabilized by 0.1% Triton X-100 and stained for 1 h at room temperature with a mouse monoclonal antibody against *P. gingivalis* ATCC 33277 NDK (1:1000; BoreDa, South Korea). The stained cells were washed and incubated for 1 hour at room temperature with Alexa Fluor 488 conjugated secondary goat anti-mouse polyclonal antibody (1:1000; Invitrogen). For labeling of actin-cytoskeleton phalloidin-tetramethylrhodamine B isothiocyanate (TRITC) was used at a dilution of 1:2000 (Sigma-Aldrich). Coverslips with fixed cells were mounted onto Corning glass microscopy slides (Corning) using VectaShield mounting medium containing DAPI (Vector Laboratories). Images were acquired using Zeiss AxioImager A1 epifluorescence microscope using QImaging MicroPublisher 3.3 cooled microscope camera and QCapture software.

### ATPase activity assays

ATPase assays were performed on cell-free culture media of GECs at 12 h post infection with *P. gingivalis* ATCC 33277. Cell-free culture media of uninfected GECs was used as negative control in this experiment. GECs were seeded in 6-well culture dishes (Corning Life Sciences DL) and infected with *P. gingivalis* at 70% confluence. 1 hour after infection specific inhibitors were added as follows: probenecid (Sigma-Aldrich) was used at final concentration of 1 mM, and methyl-β-cyclodextrin (Sigma-Aldrich) was used at a 10.5 μM final concentration. Inhibitor concentrations were determined in a set of experiments based on previous reports^59^,^60^,^61^. The *ndk*-deficient mutant strain of *P. gingivalis* was used as control for the ATPase activity. Eleven hours after addition of the inhibitors, cell culture media was collected, centrifuged (1500 × G, 5 min) and cell-free supernatants were used further in the experiment. ATPase activity was measured by Innova ATPase assay kit (Innova Biosciences) according to the manufacturer’s instructions. The experiment was performed at least three times in duplicates.

### Depletion of PNX1 by RNA interference

Cultures of primary GECs were transfected with a mixture of 10 pmol of short interfering RNA (siRNA) (Life Technologies, Silencer Select) and 3 μl/ml Lipofectamine RNAiMax reagent (Life Technologies), according to the manufacturer’s instructions. A non-targeting control siRNA was used as control (Life Technologies, Silencer Select). Forty-eight hours after transfection cells were re-fed and infected with *P. gingivalis* ATCC 33277 at an MOI of 100 for 24 h. Cell-free culture media were collected and used in NDK-specific ELISA assays. Cell monolayers were collected using 0.05% Trypsin dissociation for confirmation of successful silencing of *pnx1* on both the gene and protein levels by quantitative RT-PCR and standard SDS-PAGE western blot techniques respectively. Each experiment was performed at least three times and ELISA assays for each separate repetition were performed in duplicates.

### Measurement of NDK protein secretion by ELISA detection assays

Cell-free media of *P. gingivalis*-infected or uninfected GECs from PNX1 silencing experiments were used for the measurement of NDK. NDK-ELISA was performed as follows: cell-free media of *P. gingivalis*-infected or uninfected GECs from PNX1 silencing and ATPase activity assays experiments were diluted at a 1:1 ratio in 2x carbonate-bicarbonate buffer (Sigma-Aldrich) and coated overnight at 4 °C onto Corning disposable sterile ELISA plates (Corning). Plates were then washed and blocked with blocking buffer, containing 5% bovine serum albumin (BSA) and 4% sucrose in DPBS for 1 hour at room temperature. NDK was detected using a 1:1000 dilution of mouse monoclonal anti-*P. gingivalis*-NDK antibody (Bore Da, South Korea) in DPBS containing 5% BSA. Wells were washed three times with DPBS containing 0.05% Tween (Sigma-Aldrich) and adsorbed with 1:1000 HRP-linked goat-anti-mouse monoclonal antibody (Cell Signaling) in DPBS supplemented with 5% BSA for 1 h. This was followed by 3-times washing and subsequent incubation with 2,2′-Azino-bis(3-ethylbenzothiazoline-6-sulfonic acid) diammonium salt (Sigma) for 30 minutes at room temperature in the dark. Optical density was read at absorbance of 414 nm using a Synergy MX plate reader (BioTek Instruments). Recombinant *P. gingivalis* NDK (GenScript) was used for a standard curve at 2-fold dilutions from 2.5 to 0.08 ng/well. All assays were performed at least three times in duplicates.

### Pannexin-1 analysis by Western blotting

Cell pellets were collected from PNX1 siRNA treated or control cells by 0.05% Trypsin digestion and centrifugation at 130 × G for 5 minutes. Cell pellets were re-suspended into 100 ul of 1x Laemmle buffer (BioRad) and denatured at 95 °C for 5 minutes. The samples were loaded at 25 ug total protein/well and run on a 10% acrylamide gel, at 140 V for 1 h. Proteins were transferred onto a nitrocellulose membrane (Santa Cruz) and membranes were blocked with 5% skimmed milk in Tris-buffered saline containing 0.1% Tween-20. Proteins were detected using a PNX1-specific rabbit polyclonal primary antibody (Abcam) at 1:1000 concentration, and a horseradish peroxidase-linked goat-anti-rabbit secondary antibody (Cell Signaling) at a 1:1000 final concentration. The same membrane was further stripped and probed with anti-β-tubulin polyclonal mouse antibody (1:1000) (Invitrogen) for loading control, followed by HRP-conjugated secondary antibody (1:1000) (Cell Signaling).

### Liquid chromatography-tandem MASS-Spectrometry (LC-MS/MS) analysis

Cultures of primary GECs in 75 cm^2^ culture flasks were infected with *P. gingivalis* at an MOI of 100. Cells were collected after 2 h, 3 h or 24 h of infection and total cell proteins were extracted in protease inhibitor-supplemented RIPA buffer. Uninfected cells were used as control. *P. gingivalis*-NDK and NDK-binding proteins were concentrated using 30 ul/sample IgG beads coated with anti-*P. gingivalis-*NDK rabbit antibody. Eluted immuno-precipitated proteins from the samples were run on a regular SDS-PAGE and protein bands that were not detected in the uninfected control were submitted to LC-MS/MS analysis, performed at the Interdisciplinary Center for Biotechnology Research of the University of Florida. All MS/MS samples were analyzed using Mascot software (Matrix Science, London, UK; version 2.4.0). Mascot was set up to search IPI-Human databases assuming the digestion enzyme trypsin. Mascot was searched with a fragment ion mass tolerance of 0.8 Da and a parent ion tolerance of 10 ppm. Iodoacetamide derivative of Cys, deamidation of Asn and Gln, oxidation of Met, are specified in Mascot as variable modifications. Scaffold (version Scaffold-4.0, Proteome Software Inc., Portland, OR) was used to validate MS/MS based peptide and protein identifications. Peptide identifications were accepted if they could be established at greater than 95.0% probability and contain at least 2 identified unique peptides. Protein probabilities were assigned by the Protein Prophet algorithm^35^,^36^. The most biologically significant result was selected at the most rigorous settings possible in the Scaffold software, representing 95% or higher specificity and over 99.9% threshold of detection, at a setting of no less than 5 matching peptides for each detected protein.

### Co-localization analysis

Co-localization analysis between NDK and Myosin-9 was performed as described previously^62^. Briefly, GECs were infected with *P. gingivalis* for 6 h or 24 h at an MOI of 100 and fixed with methanol. NDK molecules were labelled using 1:1000 or 1:2000 dilution of mouse monoclonal anti-*P. gingivalis*-NDK antibody (Bore Da, South Korea) and 1:1000 dilution of AlexaFluor488-linked goat-anti-mouse monoclonal antibody (Invitrogen). Myosin-9 was labelled using a 1:100 dilution of rabbit monoclonal anti-human Myosin-9 antibody (Abcam) and 1:1000 dilution of secondary AlexaFluor594-linked goat-anti-rabbit monoclonal antibody (Invitrogen). Co-localization analysis was carried out using the co-localization analysis tool under WCIF NIH ImageJ software. The co-localization rates were measured based on Manders’ coefficient, which varies from 0 to 1. A coefficient value of zero corresponds to non-overlapping images while a value of 1 reflects 100% co-localization between the images being analyzed.

### Statistical analysis

Two-tailed Student’s *t*-test was used to calculate the statistical significance of the experimental results between two conditions (significance considered at P < 0.05).

## Additional Information

**How to cite this article**: Atanasova, K. *et al.* Nucleoside-Diphosphate-Kinase of *P. gingivalis* is Secreted from Epithelial Cells In the Absence of a Leader Sequence Through a Pannexin-1 Interactome. *Sci. Rep.* **6**, 37643; doi: 10.1038/srep37643 (2016).

**Publisher’s note:** Springer Nature remains neutral with regard to jurisdictional claims in published maps and institutional affiliations.

## Supplementary Material

Supplementary Figure 1

Supplementary Figure 2
