# Supplementary material for: Nucleoside-Diphosphate-Kinase of P. gingivalis is Secreted from Epithelial Cells In the Absence of a Leader Sequence Through a Pannexin-1 Interactome
Source: Sci Rep. 2016 Nov 24;6:37643. doi: 10.1038/srep37643 (PMC5121656; doi:10.1038/srep37643)
Supplement: Supplementary Figure 1 [file srep37643-s1.pdf]

SREP-16-21272A

Nucleoside-Diphosphate-Kinase of *P. gingivalis* is Secreted from Epithelial Cells In the Absence of a Leader Sequence Through a Pannexin-1 Interactome

Kalina Atanasova[1], Jungnam Lee[1], JoAnn Roberts[2], Kyulim Lee[1], David M Ojcius [3] and Özlem Yilmaz[2,4*]

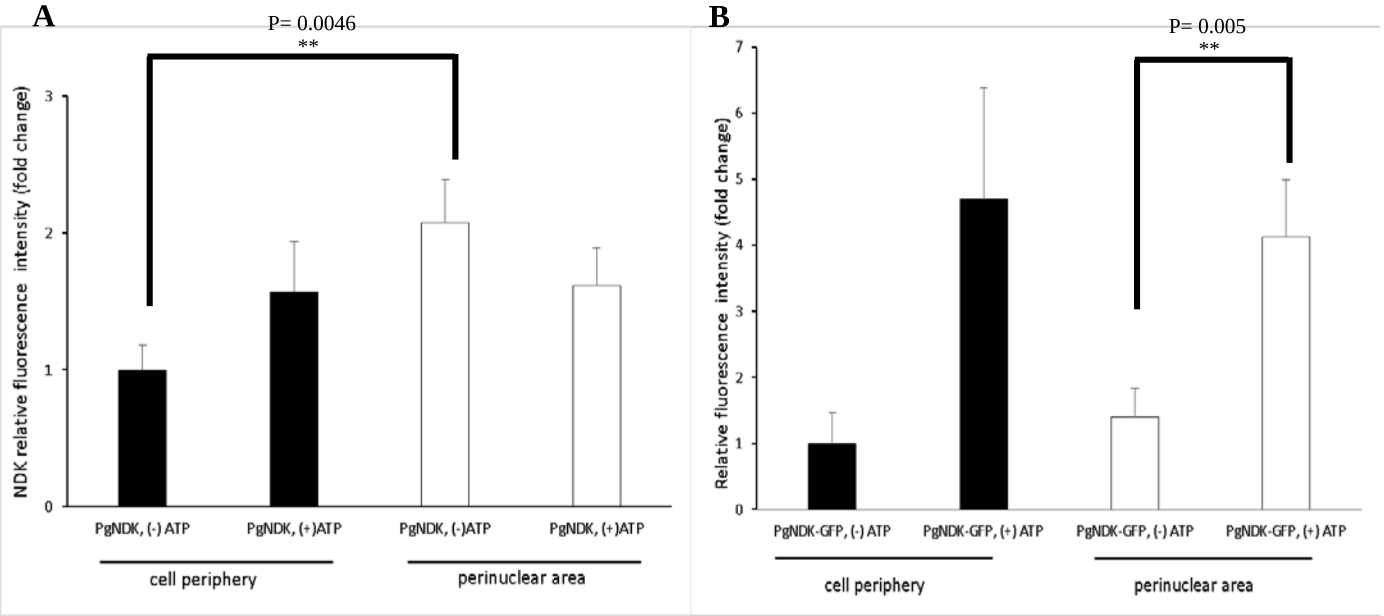

Supplementary Figure 1. Relative fluorescence intensity of green-fluorescent signal-labelled *P. gingivalis*-NDK protein in GECs. (A). *P. gingivalis*-NDK protein distribution in the perinuclear area and the peripheral cytoplasmic area of *P. gingivalis*-infected GECs, in the presence or absence of stimulation with 3mM ATP. *P. gingivalis*-NDK protein was detected using rabbit anti-*P. gingivalis* NDK antibody and visualized with anti-rabbit AlexaFluor488 secondary antibody. (B) The fluorescence intensity of expressed GFP-linked *P. gingivalis*-NDK construct in the perinuclear versus peripheral cytoplasmic area of transfected GECs in the presence or absence of stimulation with 3mM ATP. Cell boundaries were determined by the actin labeling with phalloidin-TRITC. Corrected total cell fluorescence was calculated and measurements were normalized to the mean intensity of the peripheral cytoplasm of *P. gingivalis*-infected non-stimulated cells (for A) or the *P. gingivalis* NDK-GFP transfected non-stimulated cells (for B). NDK protein was found in greater amounts in the perinuclear area in non-ATP stimulated cells, whereas a translocation to the cell periphery was observed upon stimulation with ATP, in the infected cells. ** represent P-values <0.01. Select exact P-values are also shown.
