# Supplementary material for: Nucleoside-Diphosphate-Kinase of P. gingivalis is Secreted from Epithelial Cells In the Absence of a Leader Sequence Through a Pannexin-1 Interactome
Source: Sci Rep. 2016 Nov 24;6:37643. doi: 10.1038/srep37643 (PMC5121656; doi:10.1038/srep37643)
Supplement: Supplementary Figure 2 [file srep37643-s2.pdf]

SREP-16-21272B

Nucleoside-Diphosphate-Kinase of *P. gingivalis* is Secreted from Epithelial Cells In the Absence of a Leader Sequence Through a Pannexin-1 Interactome

Kalina Atanasova[1], Jungnam Lee[1], JoAnn Roberts[2], Kyulim Lee[1], David M Ojcius [3] and Özlem Yilmaz[2,4*]

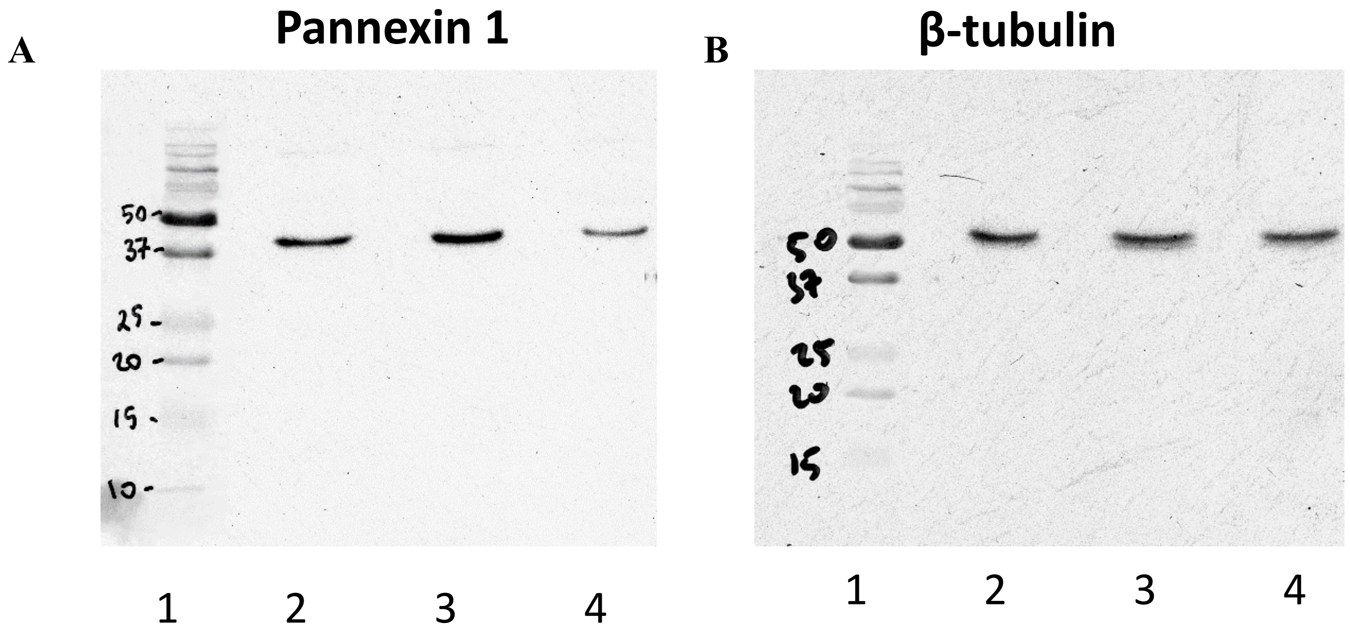

Supplementary Figure 2. Full-length Western-blot images of the data presented in the Figure 3A **(A).** 45kDa Pannexin 1 (PNX1) protein is detected using a PNX1-specific rabbit polyclonal primary antibody (Abcam, catalog #: ab124131) at 1:1000 concentration, and a horseradish peroxidase-linked goat-anti-rabbit secondary antibody (Cell Signaling) at a 1:1000 final concentration. **(B)** β-tubulin (51kDa) was used as loading control. The same membrane was further stripped and probed with anti-β-tubulin polyclonal mouse antibody (1:1000, catalog #: 322600) (Invitrogen) for loading control, followed by HRP-conjugated secondary antibody (1:1000) (Cell Signaling). Results were visualized using an enhanced chemiluminescence detection kit (Amersham Biosciences). Lane 1 indicates molecular weight marker. Lane 2, 3 and 4 indicate; untransfected (control), Non-target siRNA, and PNX1 siRNA samples respectively.
